# A Highly Sensitive TDLAS-Based Water Vapor Isotopes Sensor Using a Quantum Cascade Laser

**DOI:** 10.3390/s25030840

**Published:** 2025-01-30

**Authors:** Wenling Jin, Nailiang Cao, Yufei Ma

**Affiliations:** 1 National Key Laboratory of Laser Spatial Information, Harbin Institute of Technology, Harbin 150001, China; jinwenling@stu.hit.edu.cn; 2 Anhui Institute of Optics and Fine Mechanics, Hefei Institute of Physical Science, Chinese Academy of Sciences, Hefei 230031, China

**Keywords:** stable isotopes, atmospheric water vapor, laser absorption spectroscopy

## Abstract

Based on tunable diode laser absorption spectroscopy (TDLAS), a water isotopes detection system was developed to detect the isotopic abundance of water vapor in the atmosphere. A single 1483.79 cm^−1^ quantum cascade laser (QCL) and a 3120 cm optical path multi-pass cell (MPC) were adopted in the detection system. The selected spectral range, as well as the laser technology used, is particularly interesting for the real-time monitoring of water vapor isotopes in the atmosphere. In this study, a single laser can be used to perform high-sensitivity, rapid investigations of H_2_O, H_2_^18^O, H_2_^17^O, and HDO absorption lines. Finally, we measured the abundance values of three isotopes of water vapor in the atmosphere and compared them with data from the Global Network of Isotopes in Precipitation (GNIP) website, dedicated to exploring the possibility of in situ monitoring of H₂O isotopes in the atmosphere.

## 1. Introduction

The precise measurement of stable isotope ratios of water, HDO, H_2_^18^O, H_2_^17^O and H_2_O, is critical in a wide range of research fields, such as environmental monitoring, ecological processes and contamination source tracing [1,2,3,4]. These ratios provide information not accessible through gas concentration measurements alone. Interest in measuring the D/H, ^18^O/^16^O, and ^17^O/^16^O ratios of environmental water simultaneously is increasing. The focus stems from the information provided by the equilibrium and kinetic fractionation effects of water isotopes, which are caused by evaporation, condensation, photosynthesis, plant respiration, and the circulation between water and different substances in the lithosphere, atmosphere, and biosphere [5,6]. For example, the measurement of ^17^O enables the acquisition of new scientific insights into mass-independent and evaporative processes [7]. In the case of liquid/vapor equilibrium, the fractionation effect leads to the varied distribution of water isotopes across different circles, altitudes, latitudes and types of vegetation [8]. Therefore, quantifying the stable isotopes of hydrogen and oxygen in water has the potential to trace the atmospheric water cycle. Additionally, water vapor is the primary energy carrier in the atmosphere and plays a crucial role in regulating planetary temperature as an absorber and emitter of radiation. The accurate measurement of the stable isotopes of water is of great importance for monitoring the atmospheric greenhouse effect [9]. Furthermore, tracking the stable isotopes of water to determine the presence of water or water ice on the Moon, Mars, and Mercury can provide a critical reference for humans to expand the boundaries of space exploration and utilize space resources [10,11].

Since the 1980s, with the rapid development of isotope ratio mass spectrometry (IRMS) technology, the application field of stable isotopes has been widely expanded. Although IRMS instruments can achieve high measurement accuracy, it is still challenging that traditional analytical tools for measuring isotope ratios in water are far more cumbersome and less accurate compared to those for other molecules. This is mainly because the commonly used IRMS technique is not suitable for condensable gases. Although modern commercial IRMS instruments can achieve impressive accuracy and fast sample processing speeds, they still require complex pretreatment when analyzing water, resulting in a lengthy analysis process [12]. So far, IRMS has been used in combination with “offline” methods that convert H_2_O into H_2_ and O_2_ for mass spectrometry. Meanwhile, “offline” methods such as condensation require a sample with a large water flux typically ranging from tens to hundreds of milliliters of water vapor, which means that a large volume of air must be sampled at high altitudes due to the lower water vapor mixing ratio [13,14,15]. Additionally, the commonly used IRMS technique cannot distinguish isotopes of the same mass, such as HDO and H_2_^17^O. Therefore, IRMS cannot meet the continuous, fast, and online requirements for atmospheric environment measurements and field analysis.

Laser spectroscopy-based gas detection has the merits of high sensitivity, high selectivity, and online measurement [16,17,18,19,20,21,22]. Isotope ratio laser spectroscopy (IRLS), without complex preprocessing, has sufficiently high spectral resolution and is highly selective for molecular species and the specific isotopes, which is suitable for the applications that require real-time and rapid isotope analysis. Over the past few decades, there have been numerous reports on the isotope ratios of atmospheric components using laser spectroscopy techniques. For instance, methods employing Cavity Ring-Down Spectroscopy (CRDS) and Off-Axis Integrated Cavity Output Spectroscopy (OA-ICOS) are progressively being adopted for the continuous measurement of the isotopic ratios of gases such as CO_2_, H_2_O, and CH_4_ in the atmosphere [23,24,25]. The latest analysis systems utilizing CRDS technology achieve an accuracy of ± 0.5‰ for δ^18^O values and ± 1.5‰ for δ^2^H values [26], while another analysis system based on OA-ICOS has also been developed to achieve similar accuracy [27]. Despite the high sensitivity of OA-ICOS and CRDS sensors, these methods require complex cavities, which always need meticulous optical alignment [28,29,30] and frequent cavity reflectivity calibration [31]. Factors such as temperature and pressure can significantly affect the reflectivity of the cavity and the stability of the optical path, ultimately compromising measurement accuracy [25,32], making the practical field applications highly constrained.

Without complex and expensive instruments, TDLAS alters the laser wavelength by adjusting the injection current and temperature of the semiconductor laser, enabling the scanning of specific molecular absorption spectra. In contrast to CRDS and OA-ICOS, the mirrors inside the TDLAS system are less susceptible to contamination and do not require any locking [33,34,35]. It has been widely used in industrial, medical, environmental monitoring, and other fields due to its exceptional sensitivity, precision, selectivity, and rapid responsiveness. The first application of TDLAS to measure the ratio of water vapor isotopes was developed by Webster et al. [36]. C. Dyroff et al. have employed a TDLAS-based sensing system to measure water isotopes, which, however, only allows for measurements of δ^2^H [37]. Then, the measurement of three water vapor isotopes in situ using TDLAS with near-infrared laser diodes was proposed by T. Le Barbu et al. Due to the limited tunability range of the NIR diode laser, two lasers were employed to detect the molecular transitions of water and its isotopes (H_2_O, HDO, H_2_^18^O, and H_2_^17^O) [38]. By tuning the temperature of a single laser with a central wavelength of 2.64 μm, G. Durry et al. further determined the measurements of the molecular transitions of three H_2_O isotopes: HDO, H_2_^18^O, and H_2_^17^O, but this means that accessing all absorption lines remains quite challenging due to the time required for varying the laser temperature [39]. X. Cui et al. developed a compact isotope ratio sensor at 2.7 μm for the simultaneous measurement of the D/H, ^18^O/^16^O, and ^17^O/^16^O isotope ratios in glacier water. However, this band of selected molecular vibrational transition absorption is not suitable for measuring atmospheric water vapor due to interference caused by the spectral overlap of carbon dioxide [40]. Additionally, multiple reflections of the injected laser beam tend to generate interference fringe noise, making it challenging to ensure a satisfactory optical path length. Therefore, in order to achieve highly precise TDLAS measurements of water vapor isotopic abundance, improving the signal-to-noise ratio (SNR) of the output signals is imperative.

In this paper, a compact ultra-high sensitivity IRLS sensor capable of measuring multiple isotope ratios in the atmosphere is reported. The mid-infrared absorption band with strong absorption line strength and high laser output power is used as the measurement window for water vapor isotopes, especially δ^17^O values, which can be used to improve our understanding of various hydrological and meteorological processes (e.g., differentiate equilibrium and kinetic fractionation). This work presents a methodology that employs laser beam shaping facilities in conjunction with a broad-scan and narrow-linewidth laser to accurately measure the absorption spectra of multiple isotopes in the mid-infrared using one single laser, thereby reducing the volume of the sensing system. With the developed beam focusing system for mid-infrared sources, an equivalent optical path of 3120 cm is achieved with a sufficiently suppressed optical interference in Herriott multi-pass cell, which further improves the detection limit of such sensors. The accuracy and long-term stability of the multiple isotope ratio system are investigated by experimental measurements. Ultimately, the sensor was validated for monitoring the abundance variation of HDO as a representative example in a field application.

## 2. Detection Principle and Absorption Line Selection

### 2.1. Isotopic Abundance Measurements

Isotope ratio measurement is a straightforward and complementary approach for investigating the various processes involved in atmospheric H_2_O [41,42]. The isotope ratio *R* is generally defined as the ratio of the heavy isotope abundance to the light isotope abundance in a molecular species [43]:

(1) R=Xix/Xia

where *x* and *a* are represented as heavy isotope species (H_2_^17^O, H_2_^18^O, or HDO) and a light isotope component (H_2_^16^O), respectively. $X_{i}$ is the mole fraction of the absorbing molecules.

The relative abundance of light isotopes is several orders of magnitude higher than that of heavy isotopes. Consequently, the natural variation in *R* is generally small, making it difficult to analyze and compare differences in heavy isotope abundances, as these are often overshadowed by the more significant differences in light isotope abundances. Therefore, the *δ* deviation from the reference material is utilized to represent the isotopic composition of the sample via the following formula:

(2) $$\delta (E)‰=(R_{Sa}/R_{St}-1)\times 1000$$

where *R_Sa_* and *R_St_* represent the isotope ratio of the sample to be measured and the isotope ratio of the standard material.

The stable isotope analysis conducted internationally typically involves expressing the relative difference in abundance ratios in permille between the water isotope and the international standard reference Vienna Standard Mean Ocean Water (VSMOW), denoted by the *δ*-value:

(3) $$\delta (E)‰=\frac{R_{Sa}-R_{VSMOW}}{R_{VSMOW}}\times 1000$$

where, *R_VSMOW_* = (155.76 ± 0.05) × 10^−6^ for ^2^H, (2005.20 ± 0.43) × 10^−6^ for ^18^O and (373.00 ± 15.00) × 10^−6^ for ^17^O [44].

The laser absorption spectroscopy technique tunes the wavelength of a single-mode diode laser to the rotation–vibration transition of the molecule under investigation by adjusting the current or temperature of the diode laser. The laser beam passes through the target gas in the atmosphere and is absorbed in situ by the ambient molecules. The spectroscopic absorbance *α*(*ν*) of gas molecules can usually be expressed using the Beer–Lambert law:

(4) $$\alpha \left(\nu \right)=-\ln(I\left(\nu \right)/I_{0}\left(\nu \right))=S\left(T\right)\varphi_{\nu }PX_{i}L$$

where *I*(*ν*) is the transmitted light intensity; *I*_0_(*ν*) is the incident light intensity; *S*(*T*) is the line strength; *X_i_* is the mole fraction of the absorbing species *i*; *P* is the total gas pressure, and *L* is the absorption optical path length. The line-shape function *φ*(*ν*) characterizes the relative variation in the spectral absorption coefficient with frequency, and the integral absorption area *A* can be written as follows:

(5) $$A=\int \alpha \left(\nu \right)d\nu =PX_{i}LS\left(T\right)/n\int \varphi_{\nu }d\nu =PX_{i}LS\left(T\right)/n$$

where *n* is the abundance of isotopes predicted by the HITRAN 2020 database [45]. Combining Equation (1), the gas stable isotope ratio is given as follows:

(6) Rx=AxAa×Sa/naSx/nx

With knowledge of the absorption line strength (ascertained by the HITRAN 2020 database, for instance), the integrated absorbance *A* can be used to determine the isotopic *δ*-value relative to the water isotopic composition of the international standard material known as VSMOW.

To further analyze the impact of uncertainties in individual variables on the final results, the uncertainty of δ(E) is estimated using the arithmetic synthesis error propagation formula. Based on Equation (2), we derive the following expression:

(7) $$\delta (E)=(R_{Sa}/R_{VSMOW}-1)=f(R_{Sa},R_{VSMOW})$$

where $f(R_{Sa},R_{VSMOW})$ represents a function of $R_{Sa}$ and $R_{VSMOW}$, and the error propagation formula for δ(E) can be expressed as:

(8) $$△\delta (E)=\sqrt{(\left|\frac{\partial f}{\partial R_{Sa}}\right|△R_{Sa}{)}^{2}+(\left|\frac{\partial f}{\partial R_{VSMOW}}\right|△R_{VSMOW}{)}^{2}}$$

where

(9) $$\frac{\partial f}{\partial R_{Sa}}=\frac{1}{R_{VSMOW}},\frac{\partial f}{\partial R_{VSMOW}}=-\frac{R_{Sa}}{R_{VSMOW}^{2}}$$

Further calculation for the value of $△R_{Sa}$ is required. Since $R_{Sa}$ is equivalent to Rx, it follows from Equation (6) that:

(10) RSa=Ax⋅Sa⋅nxAa⋅Sx⋅na=g(Ax,Sa,nx,Aa,Sx,na)

where g(Ax,Sa,nx,Aa,Sx,na) represents a function related to Ax,Sa,nx,Aa,Sx and na. The error propagation formula for $R_{Sa}$ can be expressed as:

(11) △RSa=(∂g∂Ax△Ax)2+(∂g∂Sa△Sa)2+(∂g∂nx△nx)2+(∂g∂Aa△Aa)2+(∂g∂Sx△Sx)2+(∂g∂na△na)2

where

(12) ∂g∂Ax=−Sa⋅nxAa⋅Sx⋅na, ∂g∂Sa=−Ax⋅nxAa⋅Sx⋅na, ∂g∂nx=−Ax⋅SaAa⋅Sx⋅na∂g∂Aa=−Ax⋅Sa⋅nxA2a⋅Sx⋅na, ∂g∂Sx=−Ax⋅Sa⋅nxAa⋅S2x⋅na, ∂g∂na=−Ax⋅Sa⋅nxAa⋅Sx⋅n2a

Since the ratios Sa/na and Sx/nx in Equation (6) can be accurately determined by a calibration using isotope reference material, their contributions to the combined uncertainty are not considered in this study. Therefore, the expression for $△R_{Sa}$ can be obtained as follows:

(13) △RSa=(∂g∂Ax△Ax)2+(∂g∂Aa△Aa)2

Then, △δ(E) can be expressed as:

(14) $$△\delta (E)=\sqrt{(\frac{1}{R_{VSMOW}}△R_{Sa}{)}^{2}+(\frac{R_{Sa}}{R_{VSMOW}^{2}}△R_{VSMOW}{)}^{2}}$$

### 2.2. Absorption Line Selection

**Figure 1:**
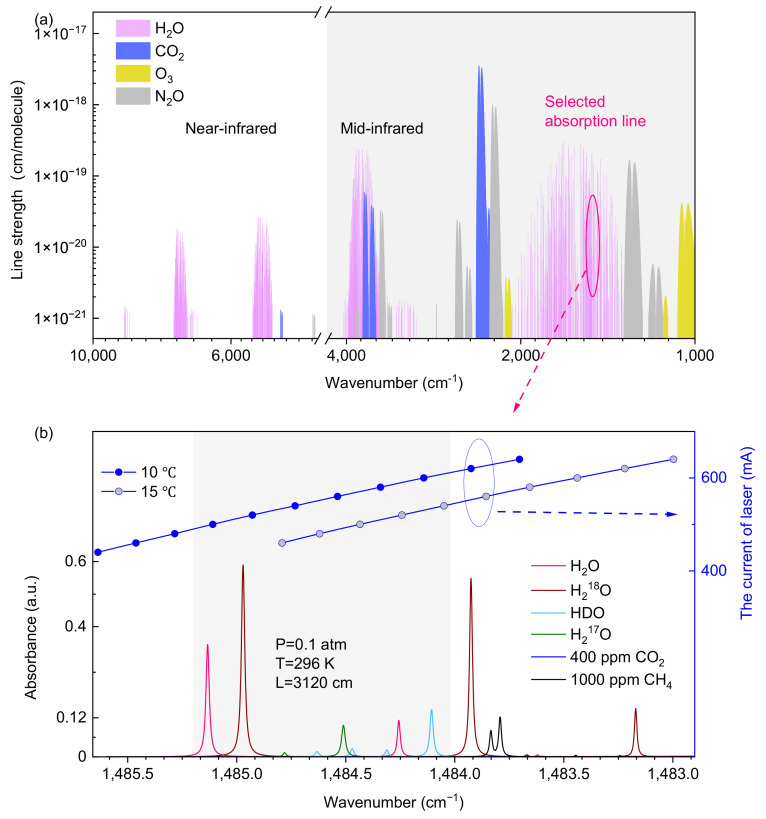
(**a**) Simulation of line strength of water vapor and main components in the atmosphere at 1000–10000 cm^−1^; (**b**) The simulated absorption lines of 2% H_2_O and the natural abundance of H_2_^18^O (2.0000×10^−3^), HDO (3.1069×10^−4^) and H_2_^17^O (3.7188×10^−4^) in the background of 400 ppm CO_2_ and 1000 ppm CH_4_ in the range of 1483.0–1485.5 cm^−1^ and the wavenumber–current characteristics of the selected laser in this range. The shaded area represents the wavenumber range while the laser is operating during the measurement of water and its isotopes.

To simultaneously measure the concentration of H_2_O and its three isotopes in the atmosphere, it is crucial to consider the following two points. Firstly, the selection of the absorption intensity and position of the target molecular absorption lines determines the measurement sensitivity and precision. The transition distance of the absorption peaks to be measured should be within the tuning range achieved by scanning the laser current at a fixed temperature. Secondly, it is necessary to consider the influence of other gases in the atmosphere on the measurements of H_2_O and its isotopes. According to the HITRAN 2020 database shown in Figure 1a, the strong line strength and spectral overlap of H_2_O and its isotopes in the mid-infrared band facilitate simultaneous, highly sensitive detection of multiple isotopes using one single laser. Considering that the commonly used 3500–4000 cm^−1^ wavenumber range for measuring water isotopes is prone to interference from major atmospheric gases, the quantum cascade laser in the 1000–2000 cm^−1^ spectral range is an optimal choice due to its advantages of continuous-wave (CW) output power levels and high line intensities. Therefore, a mid-infrared laser operated at 1483.79 cm^−1^ is selected as the light source, enabling the rapid measurement of H_2_O and its isotopes in the atmosphere at a constant tuning temperature.

As shown in Figure 1b, the overall tuning range achieved by adjusting the laser current at 10 °C spans from 1483.68 cm^−1^ to 1485.66 cm^−1^. The major potential interfering components in the atmosphere include 2% H_2_O, along with interference from the absorption of 400 ppm CO_2_, and 2 ppm CH_4_. Hence, a spectrum simulation is presented for the condition consisting of 2% H_2_O (and its isotopes at natural abundance) and 400 ppm CO_2_, with a pressure of 0.1 atm, a temperature of 296 K, and an optical length of 3120 cm. The absorption spectrum simulation for 1000 ppm CH_4_ is also provided due to its characteristic absorption near the selected water isotope peaks, to facilitate the evaluation of the calibration-free ability of the developed sensor with CH_4_ standard gas as an alternative to water vapor. The circled pair of lines represents the operating conditions of the laser during the measurement of the CH_4_ standard gas. Note that the vapor pressure inside the cavity is set at 0.1 atm to ensure high measurement quality by minimizing the effects of the water–vapor mixing ratio on spectral broadening and the signal-to-noise ratio. The line strengths of H_2_O, HDO, H_2_^18^O, and H_2_^17^O exhibit comparable magnitudes within this laser tuning range. In our experiment, four adjacent lines, including a HDO line at 1484.11 cm^−1^, a H_2_^17^O line at 1484.51 cm^−1^, a H_2_^18^O line at 1484.97 cm^−1^, and a H_2_O line at 1485.13 cm^−1^, are well separated within a relatively narrow spectral region. Consequently, the high-resolution absorption spectra can be accurately measured without interference from other atmospheric gases.

## 3. Sensor Configuration

### 3.1. Experimental Setup

**Figure 2:**
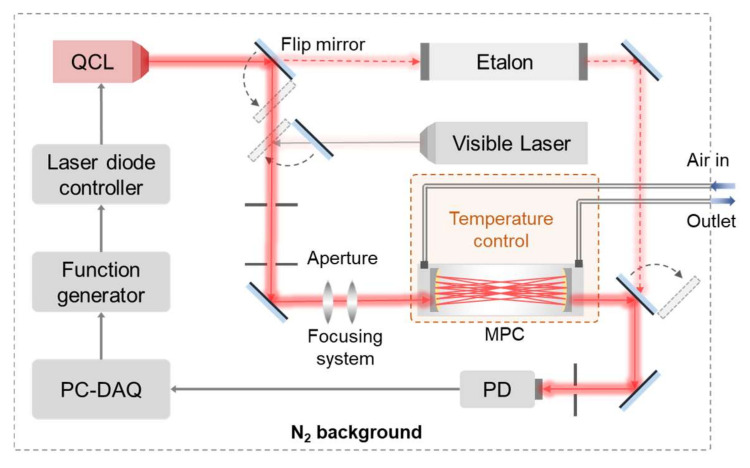
Schematic of a CW-QCL-based sensor system for the detection of four trace isotopes of water vapor.

The water isotope ratio detection system in the atmosphere based on one single laser is depicted in Figure 2. According to the analysis of the absorption line in Section 2.2, a water-cooled CW-QCL (LPD53-033, Hamamatsu Photonics, Hamamatsu, Japan) with high laser output power in the mid-infrared band was selected. The water cooling system is home-made and this laser operates in the mid-infrared band, where H_2_O and its isotopes exhibit strong absorption lines that are closely spaced. The nominal wavelength range can reach 1478.01 cm^−1^ to 1485.64 cm^−1^ at the specified operating temperature from 10 °C to 50 °C. The temperature and current of the CW-QCL centered at 1483.79 cm^−1^ are regulated by a laser diode controller (QubeCL, ppqSense S.r.l., Sesto Fiorentino, Italy) as described. The CW-QCL is maintained at a constant temperature while a triangular wave signal, generated by a function generator with a scanning voltage amplitude of 1.5 V and a frequency of 10 Hz, is employed to scan the wavelength of the laser encompassing the absorption lines of H_2_O, H_2_^18^O, H_2_^17^O, and HDO rapidly. The laser emission is free from mode-hopping within the specified tuning range. The output radiation from the CW-QCL is directed toward a flip mirror and split into two optical paths. One of the beams propagates through a germanium etalon to characterize the relative variation in wavenumber during laser scanning mode. The other beam, used for isotope measurement, is coaxial with a visible diode beam (HNL050LB, Thorlabs, Newton, NJ, USA) through a pair of reflectors, then coupled into an MPC via a pair of beam-shaping lenses. Note that the larger divergence angle of the mid-infrared light source, as compared to the near-infrared light source, necessitates specific optimization effects which are detailed in Section 3.2. The laser beam exiting from the MPC is focused onto a thermoelectrically cooled mercury cadmium telluride (MCT) detector (PVIM-4TE-8, Vigo Photonics, Otwock, Poland). Subsequently, the absorption signals from the detector are digitized and processed by a data acquisition (DAQ) card (PCI-6356, National Instruments, Austin, TX, USA), which is controlled by a LabVIEW program running on a PC.

To mitigate the impact of water vapor adsorption and temperature fluctuations on isotopic abundance, the gas cell and its inlet and outlet gas paths are equipped with heater bands and insulation layers. The gas cell temperature is monitored using calibrated platinum resistors (Pt100), and a PID temperature controller is employed to regulate the temperature of the MPC. Additionally, considering the interference caused by the broadening of approximately 2% H₂O in the air background at atmospheric pressure, the optical system is operated within a nitrogen-filled glovebox. The pressure is dynamically balanced through the utilization of a mass flow meter and pressure controller.

### 3.2. Optimization

**Figure 3:**
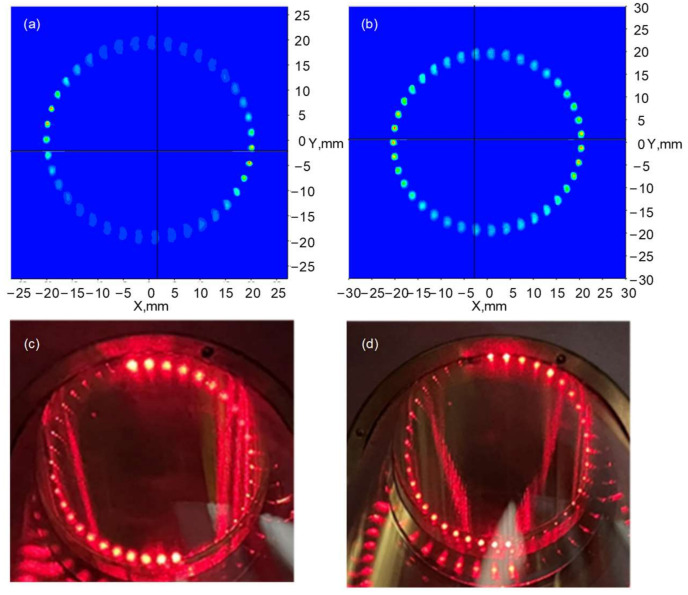
Simulation of the spot patterns on the exit mirror (**a**) without using a focusing system and (**b**) with a focusing system; photographs of the real spot patterns on the exit mirror (**c**) without using a focusing system and (**d**) with a focusing system. A red visible diode laser beam was used to visualize the spot pattern.

The MPC, with an equivalent optical path of 3120 cm, is primarily composed of two concave mirrors spaced 400 mm apart, each with a focal length of 200 mm and an outer diameter of 2 inches. The incident laser will form a dense pattern with minimal overlap on the two mirror surfaces as depicted in Figure 3. The SNR of the system is influenced by the energy density of the reflected light spots on the concave mirrors, and it depends on whether these spots overlap, which is particularly affected by mid-infrared sources with a large divergence angle. To achieve high absorption by path accumulation with minimal interference, the spot diameter on the concave mirror in MPC must be precisely controlled. Therefore, we designed a beam focusing system for a mid-infrared laser in MPC to precisely adjust the parameters of the Gaussian beam emitted by QCL. The waist position of the converged Gaussian beam is located inside the MPC, where the beam undergoes multiple convergences and reflections while accumulating optical path length, thereby optimizing the spot size on the reflective surface for the enhanced coupling efficiency.

The Gaussian beam emitted by the QCL laser has waist diameters of d_x_ = 1.859 ± 0.040 mm and d_y_ = 2.478 ± 0.040 mm. After being coupled through the focusing system, which consists of two convex mirrors with focal lengths of 500 mm and 100 mm separated by a distance of 448 mm, and passing through the input aperture of the first concave mirror of the MPC, the waist diameter reaches d_x_’ = 2.385 ± 0.040 mm and d_y_’ = 2.242 ± 0.040 mm upon reaching the second concave mirror. The distances from the focusing system to the QCL and MPC are 250 mm and 78 mm, respectively.

Based on all the determined parameters, the simulation results of the spot patterns on the exit mirror are depicted in Figure 3a,b. The utilization of the focusing system leads to an observed enhancement in power density within the spot patterns on the exit mirror. Then, a focusing system composed of two convex lenses is established to verify the designed dot pattern, as shown in Figure 3c,d, and the actual spot patterns on the exit mirror exhibit excellent agreement with those illustrated in Figure 3a,b.

**Figure 4:**
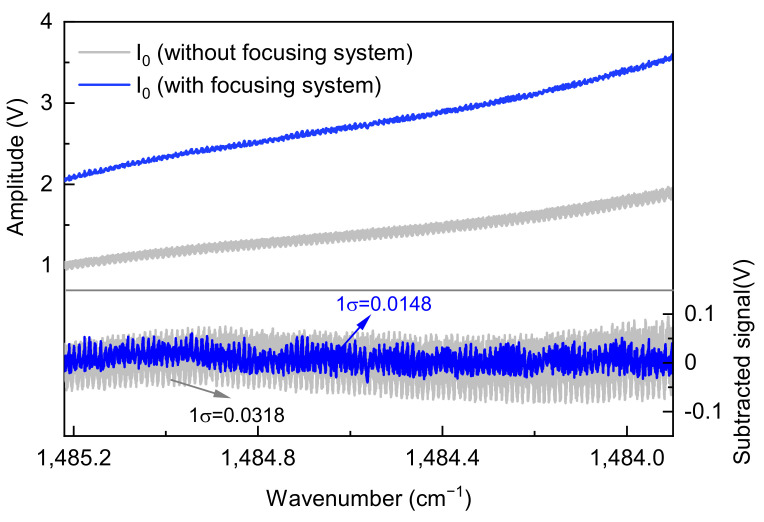
The dependence of the SNR on the focusing system suppression. The upper panel displays the raw data obtained from the PD positioned behind the MPC. The bottom panel depicts the signal after removing the baseline from light intensity.

By scanning the wavelength of the laser, the results for the beam focusing system utilized in MPC are shown in Figure 4. It is observed that, compared to the sensor system without a focusing mechanism, the sensor equipped with the focusing system exhibited a significant reduction in interference noise within the spectral signal and an increase in signal amplitude. After correcting for baseline variations due to changes in light intensity, the noise standard deviations (1σ) of the spectral signal are 0.0318 V and 0.0148 V for systems without and with the focusing system, respectively, corresponding to an SNR of 41.27 and 175.60, which indicates a remarkable improvement in system performance.

## 4. Sensor Performance

### 4.1. Calibration-Free Ability Evaluation by Measuring Concentration-Calibrated Methane Gas

**Figure 5:**
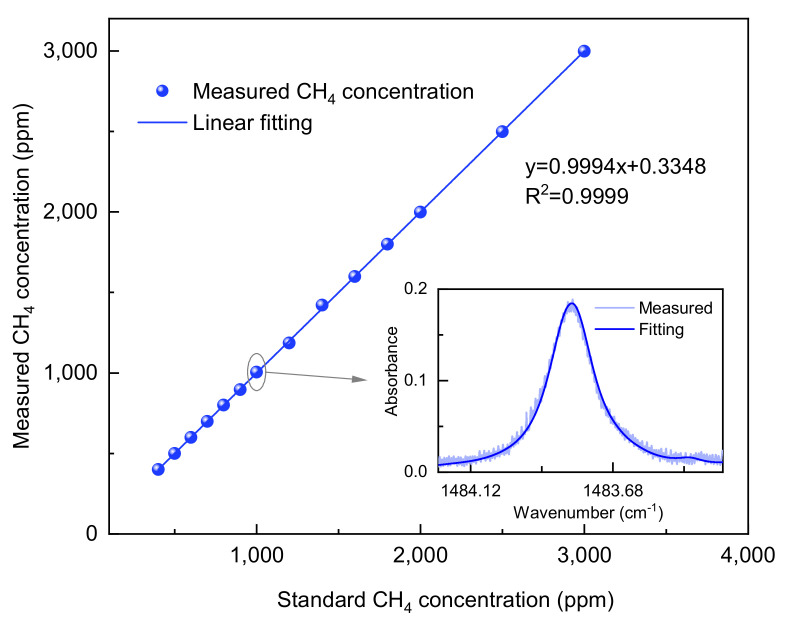
Determination of the accuracy of the concentration measured by the sensor by taking CH_4_ gas as an example. Inset: comparison of the measured and simulated absorption of 1000 ppm CH_4_ at 1 atm.

For such a highly adsorbent and ubiquitous molecule as water vapor, it is exceedingly challenging to generate a standard gas with precise concentration. Consequently, the absorption line of methane molecules at a wavenumber of 1483.79 cm^−1^, shown in Figure 1b, which can be covered within the scanning range of the same laser at 15 °C, has been chosen for evaluating the accuracy in measuring gas concentrations. With the assistance of a methane (CH_4_) standard gas and a computerized gas dilution system (Sonimix 7100, LNI Swissgas, Monteggio, Switzerland), CH_4_ gas standards with varying concentrations (ranging from 400 to 3000 ppmv) are sequentially introduced into the MPC (HC30L-M02, Thorlabs, Newton, NJ, USA), and the reliability of the established trace gas detection system is verified by a stepwise concentration measurement. To guarantee accurate measurements, the spectrum of each CH₄ gas standard is obtained by averaging 100 scans at 296 K and 1 atm. The measured absorption spectrum is fitted using a Voigt profile to obtain the integrated absorption *A*, and the characteristic absorption line of 1000 ppm CH_4_ is shown in Figure 5 (inset), which shows excellent agreement with the Voigt fitting. The concentration information derived from the average absorbance signal of CH₄ is then compared to the corresponding standard gas concentrations. The results of the linear regression analysis with the standard gas concentration and the concentration calculated by the fitting algorithm are shown in Figure 5. An R-squared value of 0.9999 is obtained, indicating that the measured concentrations of gas samples agree very well with the mixed concentration.

### 4.2. Long-Term Performance of Water Isotopes

**Figure 6:**
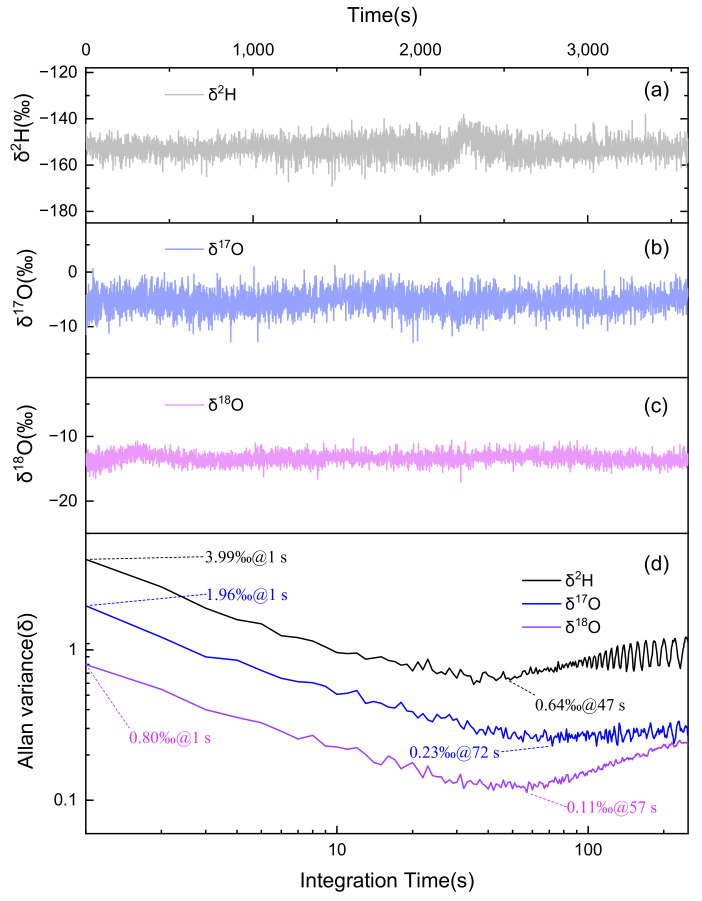
Long-term measurement results for (**a**) HDO, (**b**) H_2_^17^O, and (**c**) H_2_^18^O, and (**d**) the corresponding Allan deviation analysis.

The long-term stability and noise evaluation of the water multi-isotope sensing system were carried out using the water vapor contained in the atmosphere. Using the uncertainty calculation formula from Section 2.1 and the measurement data, the final measurement uncertainties for HDO, H_2_^18^O, and H_2_^17^O are 2.48‰, 0.67‰, and 38.36‰, respectively. The measurement uncertainty of δ(E) can reach the order of magnitude of 10⁻⁴. Therefore, we retain two decimal places in the representation of △δ(E). However, although the measurement uncertainty $△R_{Sa}$ of H₂^1^⁷O is comparable to the results of the other two isotopes, the value of △δ(E) is significantly larger. This is due to the current limitations of the VSMOW database due to the complex fractionation of H₂^1^⁷O itself. It deserves a more precise investigation in this community to enhance the performance further. To ensure optimal measurement conditions, the Allan variance analysis was performed to assess system stability, detection limits, and optimal averaging time of the detection [46]. Throughout 1 h of continuous measurement, atmospheric water vapor and its isotopes entered the MPC and were concurrently recorded by the sensing system for the *δ* value of three isotopes. The results are shown in Figure 6a–c and Allan deviation plots for HDO, H_2_^18^O, and H_2_^17^O are shown in Figure 6d. It can be observed that the Allan variance shows a trend of initially decreasing and then increasing. When “white noise” dominates, the Allan variance decreases as the averaging time increases, thereby improving the system stability. But the influence of system drift becomes more pronounced with increasing integration time, resulting in a decline in system stability. It can be seen from Figure 6d that the measurement precision of HDO, H_2_^18^O, and H_2_^17^O are 3.99‰, 0.80‰, and 1.96‰, respectively, at an integration time of 1 s. The measurement precision for HDO, H_2_^18^O and H_2_^17^O could reach 0.64‰, 0.11‰, and 0.23‰ with the best integration time of 47 s, 57 s, and 72 s, respectively. The values are all in the order of 1‰, which could meet the typical accuracy requirements for atmospheric isotopic abundance measurements.

### 4.3. Field Applications for Atmospheric Water Isotopic Abundance Detection

**Figure 7:**
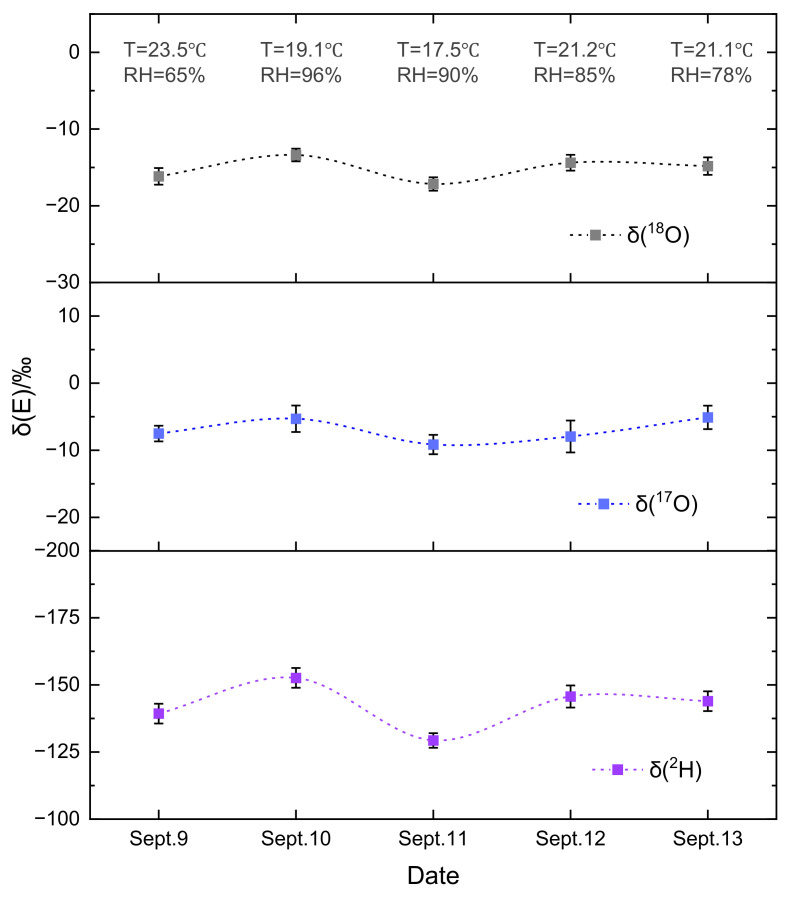
Continuously measured the abundance of water isotopes in ambient air for five days on 9–13 September 2024 in Changchun. Error bars show the 1-σ standard deviation from 30 min measurements.

The field performance of the water multi-isotope detection system was evaluated by a five-day measurement of atmospheric isotope abundance. Therefore, water vapor in ambient air is selected as a representative example for monitoring its abundance variation. The temperature and pressure inside the MPC were precisely maintained at 308 K and 0.1 atm to avoid the effects of water vapor adsorption and the broadening of adjacent absorption peaks. The sensing system is located in Changchun, Jilin Province, and the measurements are performed in a laboratory setting. Gases from the outdoor atmosphere are introduced into the MPC via a 1/4-inch polyethylene tube. The design of the nitrogen background and the temperature control system helps to prevent the influence of environmental variations on the experimental results. The experiments were conducted from 9 September to 13 September 2024, with measurements taken for 30 min at the same time each day. The data presented in Figure 7 reveal that the abundance of water isotopes remained relatively stable during this period, with average fluctuations for HDO from standard isotope deviation *δ*-value ranging from −152.61‰ to −139.32‰, H_2_^17^O from −9.14‰ to −5.11‰, and H_2_^18^O from −17.16‰ to −13.38‰, respectively.

These variations in water isotope abundance levels were clearly within the range of monitoring data provided by the corresponding sites of the Global Network of Isotopes in Precipitation (GNIP) [47], established by the International Atomic Energy Agency (IAEA) and the World Meteorological Organization (WMO), which has collected isotope data from more than 1200 stations worldwide. During each monitoring period, there were relatively minor fluctuations in water isotope abundance, with a relative standard deviation of less than 4.72‰, indicating excellent long-term operational stability.

## 5. Conclusions

In conclusion, we have demonstrated a highly sensitive mid-infrared IRLS sensor with a 31.2 m MPC that enables the rapid measurement of the abundance of three water isotopes. A CW-QCL with a wavenumber of 1483.79 cm^−1^ was employed to target the absorption lines of HDO, H_2_^17^O, H_2_^18^O, and H_2_O within a narrow spectral range of approximately 2 cm^−1^. A focusing system was designed for a mid-infrared laser, increasing the SNR of gas absorption by more than four times. The accuracy in measuring gas concentrations was initially evaluated with the assistance of a CH_4_ standard gas, yielding an R-square value of 0.9999. The sensor system was subsequently employed for the simultaneous detection of an abundance of HDO, H_2_^18^O, and H_2_^17^O using the same QCL. The system stability was evaluated by performing Allan deviation analysis, achieving an MDL of 0.64‰ for HDO, 0.11‰ for H_2_^18^O, and 0.23‰ for H_2_^17^O, at an integration time of 47 s, 57 s, and 72 s, respectively. Additionally, preliminary tests of atmospheric water vapor isotope abundance changes, performed over five consecutive days, revealed the feasibility of developing an isotope monitoring system using this laser absorption spectroscopy sensor. Unfortunately, the absorption line intensity of the molecules in existing databases is usually not accurate enough for isotopic ratio determination with high precision due to factors such as temperature, pressure, and spectral line overlap. With the precise determination of the absorption line strength in the future, the developed sensors will become a feasible choice to conventional IRMS with the advantages of being highly selective for molecular species and the specific isotopes, direct measurements on water vapor, and no sample pretreatment.

## Data Availability

The data that support the findings of this study are available on reasonable request from the corresponding author.
